# Spectral convergence in tapping and physiological fluctuations: coupling and independence of 1/f noise in the central and autonomic nervous systems

**DOI:** 10.3389/fnhum.2014.00713

**Published:** 2014-09-11

**Authors:** Lillian M. Rigoli, Daniel Holman, Michael J. Spivey, Christopher T. Kello

**Affiliations:** Cognitive and Information Sciences, University of CaliforniaMerced, CA, USA

**Keywords:** complexity matching, long-range correlations, interdependent coordination, tapping, spectral analysis

## Abstract

When humans perform a response task or timing task repeatedly, fluctuations in measures of timing from one action to the next exhibit long-range correlations known as 1/f noise. The origins of 1/f noise in timing have been debated for over 20 years, with one common explanation serving as a default: humans are composed of physiological processes throughout the brain and body that operate over a wide range of timescales, and these processes combine to be expressed as a general source of 1/f noise. To test this explanation, the present study investigated the coupling vs. independence of 1/f noise in timing deviations, key-press durations, pupil dilations, and heartbeat intervals while tapping to an audiovisual metronome. All four dependent measures exhibited clear 1/f noise, regardless of whether tapping was synchronized or syncopated. 1/f spectra for timing deviations were found to match those for key-press durations on an individual basis, and 1/f spectra for pupil dilations matched those in heartbeat intervals. Results indicate a complex, multiscale relationship among 1/f noises arising from common sources, such as those arising from timing functions vs. those arising from autonomic nervous system (ANS) functions. Results also provide further evidence against the default hypothesis that 1/f noise in human timing is just the additive combination of processes throughout the brain and body. Our findings are better accommodated by theories of complexity matching that begin to formalize multiscale coordination as a foundation of human behavior.

## Introduction

All behaviors of biological organisms can be viewed as phenomena of coordination, including human behaviors. Neurons work together to create temporal patterns of neural activity, and those patterns play important roles in motor activities leading to overt human behaviors. Likewise, behaviors result in changes to sensory and proprioceptive inputs that affect patterns of neural activity. Thus coordination happens amongst the components of brains and bodies, and also between brains, bodies, and their environments.

Perhaps the most fundamental expression of coordination in human behavior is found in the relative timing of events. Movements of the eyes must be timed relative to those of the hands to draw a picture (Huette et al., 2013); movements of hands must be timed relative to those of the vocal tract to gesture during speech (Kelly et al., 2010); and movements of the legs must be timed with movement of the ball in soccer (Bartlett et al., 2007), just to name a few examples. These are all exquisite phenomena of timing and coordination, but it is often useful to study simpler cases to formulate basic principles and theories.

From this perspective, one of the simplest cases of coordination occurs when brief, individual behaviors are timed in direct relation to clearly delineated events in the environment—stepping on the gas or brake pedal in response to a traffic light, for instance, or working on an assembly line to perform the same action repeatedly on each widget being transported along a conveyor belt. The experimental analogs to these illustrative examples are simple response times to individual stimuli, and tapping in time with a metronome. These experimental paradigms have been used in thousands of studies, with response times figuring most prominently in experimental psychology (Holden et al., 2009), and tapping in motor control (Rosenbaum, 2009).

Despite the intimate relationship between timing and coordination, theoretical approaches to response times and tapping times do not usually refer to coordination. Instead, response times are usually studied in terms of information processing, where time is theorized to reflect the number of processing steps needed to identify each stimulus, and then choose and prepare a response (Sternberg, 1998). Tapping times are usually studied in terms of timing mechanisms (Georgopoulos, 2000), where questions focus on the nature of internal clocks and their associated neural machinery.

### The puzzle of 1/f noise

Studies of information processing and clocks have led to many advances in theories of cognitive and neural processes for decades, and this progress is likely to continue for some time. However, a general property of response times and tapping times has been established over the years, and it is not easily explained within these paradigms. Responses and taps vary from one to the next, even when there is no overt change in stimuli, the metronome, or any other task conditions. This is not surprising in itself, given that humans are not robots or machines in any traditional sense of the words. One should expect a certain amount of imprecision in human timing that could be described as “noise”.

The puzzling property concerns the kind of noise observed in human timing, and many other fluctuations in biological and complex systems. A default assumption of most statistical models used in experimental psychology is that noise (i.e., error variance) in repeated measures is Gaussian and uncorrelated. The term “uncorrelated” means that current and previous measured values of noise provide no information about future measurements. This simplifying assumption is useful for statistical models, but we know it is incorrect because memory is universal to all human and biological systems—not memory as storage of information, but the more general sense that system states are conditioned on their past, and therefore carry some of their history forward in time.

The memory inherent to human and biological systems suggests that noise in human timing should be correlated in some way. For instance, homeostatic systems are often expected to exhibit negative correlations in their fluctuations, as a result of negative feedback. When synchronizing to a metronome, if one tap falls behind the beat, the next tap would be adjusted earlier in time, and vice versa. One can measure such negative feedback by taking a time series of tap intervals, and a copy of the same time series where values are shifted backward in time, and then correlating the time series with itself at different lags. Such *auto-correlation* analyses show evidence to support the presence of negative correlations in tapping data (Wing and Kristofferson, 1973), but negative correlations account for only a small amount of the noise variance. Most of the noise in human response times and tapping times exhibits positive auto-correlations (Pressing and Jolley-Rogers, 1997).

In general, positive auto-correlations can be understood in terms of hysteresis—simply put, systems are sluggish to change their states. For instance, if a response is relatively fast on one trial, then whatever system conditions caused the fast deviation will still be in play on the next trial, at least to some degree. This principle can explain positive auto-correlations in general, but it is the specific pattern of auto-correlations that is puzzling. In particular, they decay slowly as an *inverse power law* function of increasing lag, *C*(*k*)~1/*k*^λ^, where *C*() is the auto-correlation function, *k* is the integer lag, and *λ* is the power law exponent. This kind of positive auto-correlation is known as *long-range correlation* because the power law indicates that *all* past states play a role in determining any given current state. It is also known as *1/f noise* because the auto-correlation function can be expressed in the frequency domain as a relation between spectral power and frequency, *P*(*f*)~1/*f^α^*, where *P*() is the spectral function, *f* is frequency, and *α* is the power law exponent. Exponents estimated from timing data have varied across studies and conditions, but most estimates have been near 1.

The presence of 1/f noise has been reported in many studies of human response times (Van Orden et al., 2003) and tapping times (Ding et al., 2002), as well as other repeated measures of human behavior (Gilden, 2001) and neural systems (Allegrini et al., 2009). These reports have stirred up much debate. Some of this debate has concerned the veracity of findings (Farrell et al., 2006), with opponents arguing that observed auto-correlations actually may not be long-range but short-range instead (i.e., fall off exponentially with lag, instead of an inverse power law function). However, recent studies have compared these two statistical models and found 1/f noise to better account for the data (Gilden, 2009).

Accepting that the noise in human timing follows a 1/f scaling relation, most of the debate has focused on theoretical explanations. One reason for debating 1/f noise is that the theoretical constructs of clocks and information processing yield no ready insights in and of themselves. Certainly one can add mechanisms to information processing models and clock models that exhibit 1/f noise, and this has been done (Torre and Wagenmakers, 2009). Perhaps the most general mechanism thus far has been *strategy* *shifting* (Diebold and Inoue, 2001), whereby a perturbation is added to each response time or tapping time that reflects discrete shifts among distinct plateaus in response strategies. The varying duration of these plateaus, and non-stationarity of shifting among them, has been shown to yield 1/f noise under certain parameterizations.

One problem with strategy shifting and similar accounts is that they appear *post hoc*, in that they do not provide principled answers as to why information processing and clock models would include such processes. Another problem is that such domain-specific processes are difficult to generalize to other repeated measures of human activity that exhibit 1/f noise, such as speech acoustics (Kello et al., 2008) and affect ratings (Delignières et al., 2004), and repeated measures of human neural activity as well (Linkenkaer-Hansen et al., 2001). We believe that progress will continue to be made by improving and expanding domain-specific accounts related to clocks and information processing, but here we investigate two domain-general accounts aimed at the broader range of 1/f phenomena in human and biological systems.

### Two domain-general accounts of 1/f noise

The generality and ubiquity of 1/f noise has led some researchers to formulate two classes of domain-general explanations: a *process* *summation* account and an *interdependent coordination* account. The process summation account is based on sums of processes across various timescales, and the interdependent coordination account is based on the interdependence of processes necessary for coordination. Here we describe each in turn, and then present an experiment designed to test these alternative accounts of 1/f noise in human timing and physiology.

Regarding the process summation account, a 1/f-like signal can be created by sampling from three or more uncorrelated noises generated over different timescales and amplitudes (with timescale inversely related to amplitude), and summing the samples together (Wagenmakers et al., 2004). A 1/f signal can be similarly created by summing independent processes whose exponential decay rates span a range of timescales (Granger, 1980). In either case, the signal will be 1/f-like only within the range of timescales sampled. Then again, 1/f noise in human behavior can be observed only within a limited range of timescales due to limits on measurement (Van Orden et al., 2005).

The human brain and body is composed of processes that unfold over a wide range of timescales, from fast ion channel dynamics to slower changes in neurotransmitters, cardiovascular, and various homeostatic processes, and even slower changes in hormones, circadian dynamics, and developmental processes in general (Bassingthwaighte et al., 1994). A similar claim can be made regarding cognitive processes, from the millisecond dynamics of perception, to the waxing and waning of attention that may span seconds to minutes, to processes of decision-making, planning, and learning that may span anywhere from seconds to years (Ward, 2002). It seems quite plausible that any measurement of human behavior may be influenced by any combination of these ongoing processes. If the magnitude of influence (i.e., amplitude) is generally inversely related to timescale, then one would expect these processes to sum up to 1/f noise in repeated measurements of response times, tapping times, and any other measure of human behavior.

The interdependent coordination account is based on interactions among system components, rather than summations of independent processes. The coordination of behavioral activity requires interactions among whatever components and events are being coordinated. The same is true for neural and physiological activities, the difference being that the components and events are different and reside on shorter spatial and temporal scales. We can say further that interdependencies among system components must strike a balance between too much and too little coupling as a result of interactions (Kello and Van Orden, 2009). Too much coupling would result in interlocked patterns of activity that are unable to differentiate or adapt to changes in conditions. Too little coupling would fail to support the emergence of coordination patterns that extend in space and time. Instead, adaptive systems exhibiting coordination need loosely coupled components that support the formation of many different potential patterns of activity.

The balance of coupling and its relationship to pattern formation has been formalized in statistical mechanics in terms of *metastability* (Kelso, 1995), and the dynamics of interactions that underlie metastability have been shown to produce 1/f noise (Usher et al., 1995). Metastability appears to be a useful property for biological and behavioral systems in general, because it endows them with an ability to respond and adapt to their ever-changing conditions (Sasaki et al., 2007; Pinder et al., 2012). On this account, 1/f noise reflects fluctuations across multiple timescales that result from patterns being organized and re-organized across multiple timescales. Thus 1/f noise should be a general property of any metastable system, including human systems involved in response times and tapping times.

This approach to 1/f noise and other power laws in nature was made famous by models of *self-organized criticality* (SOC; Bak et al., 1987). The ubiquity of power laws in nature, like 1/f noise, led physicists to hypothesize that *critical points* may be common attractors of complex systems in nature (Bak, 1996). Critical points are associated with (second-order) phase transitions in systems of many interdependent elements, where dynamics take on unique properties of memory and symmetry-breaking (Stanley, 1987). Original models of SOC were criticized as models of human behavior because they more closely resemble models of avalanches, forest fires, and other physical complex systems (Wagenmakers et al., 2005). However, a large body of work has shown how SOC may be a functional principle of neural networks and other physiological networks (see Kello, 2013).

The *interdependent coordination* account is similar to the *process summation* account, in that both provide a rationale for the ubiquity of 1/f noise. However, they make different predictions when it comes to taking multiple repeated measurements of human behavior. Kello et al. highlighted this distinction between accounts by measuring two aspects of key-press dynamics (Kello et al., 2007). Participants made repeated simple responses to series of visual cues, and both response times and key-press durations were recorded. A key-press duration is the very brief period of time (~100–150 ms ) that a key remains in contact with its sensor for a normal, ballistic keystroke. Both domain-general accounts predict 1/f noise in both time series of measurements.

The accounts differ in whether they predict the *same* 1/f signal to appear in each time series, or whether distinct 1/f signals may arise from simultaneous yet distinct measures of behavior. The process summation account predicts the same 1/f signal because key-press response times and durations should draw from roughly the same set of summed processes, especially at the larger timescales (e.g., waxing and waning of attention and circadian rhythms). The reasons are that the two measurements are inextricably paired for each keystroke, are produced by overlapping sets of muscles, and effectively occur at the same time relative to the timescales of 1/f noise spanning dozens and hundreds of responses. It is difficult to hypothesize how these measurements could tap into distinct sets of component processes spanning the same timescales as 1/f noise. By contrast, interdependent coordination holds that any given system or subsystem can exhibit 1/f noise on its own, or in coupling with other systems. The reason is that interdependence can hold for components at all scales, and criticality can create dynamics with long-range memory (i.e., correlations) for any given subsystem. In other words, 1/f noise is hypothesized to pervade the heterogeneous networks of interacting processes in human systems.

Results from four experiments reported by Kello et al. (2007) showed that key-press response times and durations were independent of each other, in terms of exhibiting 1/f noises that were uncorrelated with each other, and also in terms of independently manipulating the 1/f noise in response times while leaving key-press durations unaffected. The authors argued that the data provided evidence against the process summation account, but were consistent with the interdependent component account. However, a subsequent reanalysis of these data indicated more subtle, nonlinear relationships between the time series (Moscoso del Prado Martín, 2011). Thus while the process summation account is called into question, more experiments and analyses are needed to investigate the nature of coupling and independence among simultaneous measures of 1/f noises (e.g., Kello et al., 2008).

### Complexity matching as measured by spectral convergence

The present experiment and analyses were designed to further investigate the nature of 1/f noise in human behavior by measuring coupling based on a recently formulated theoretical principle known as *complexity matching* (West et al., 2008; Aquino et al., 2010, 2011). Theoretical analyses using statistical mechanics have shown that, when two complex systems become coupled, there is maximal rate of information exchange between them when their power laws converge. This formalization of coupling is different from more standard measures like synchronization. Complexity matching between two signals does not refer to phase relations—instead, it refers to convergence of the two power spectra. Thus coupling in terms of complexity matching means that each system retains its own distinct phase dynamics, yet the systems affect the statistical character of each other’s dynamics. This effect is equivalent to an exchange of information between two given systems, in the sense of mutual information.

Complexity matching is a theoretical construct general to all complex systems, but it has already garnered some empirical support in studies of dyadic coordination, which can be viewed in terms of informational coupling between two human complex systems. Marmelat and Delignières (2012) conducted an experiment in which each participant in a dyad swung a hand-held pendulum, with instructions to swing in synchrony. Synchronization is a direct phase relation, but deviations from synchrony were analyzed for 1/f noise. Results showed that the spectral shape of 1/f noise for each member of a dyad converged to the extent that coupling was facilitated by visual and physical contact. This convergence could not be explained in terms of simple phase relations because there were no reliable cross-correlations in the time series of deviations from synchrony. Other more recent experiments showed the same basic effect, but in the speech signals of dyads engaged in conversation (Fusaroli et al., 2013; Abney et al., 2014).

Dyadic coordination is one example of two interacting systems, but as we discussed at the outset, humans are composed of many components across many scales that must coordinate in order to function. Complexity matching suggests that the coordination of two subsystems in a single individual may manifest as a convergence in their 1/f noise spectra when repeated measures are taken. Evidence for spectral convergence in 1/f noise would provide further evidence against the process summation account, provided that this convergence was not simply a product of correlated time series. The present experiment tested this hypothesis by measuring tapping deviations and key-press durations while either synchronizing or syncopating with a metronome. Previous studies have shown slightly stronger 1/f noise in timing deviations during syncopation (Chen et al., 2001), so we varied tapping between synchronization and syncopation to test whether an effect on timing deviations would dissociate from an effect on key-press durations.

To further investigate coupling in terms of complexity matching, we wanted to compare 1/f noise in key-presses with other fluctuations in physiological activity that either were or were not responsive to the metronome. For the former, we presented a flash of light with each auditory beat of the metronome, and measured fluctuations in the pupil dilation response across audiovisual beats of the metronome. In the synchronization condition, pupil and key-press responses occurred to the same stimuli, and roughly at the same time. If this co-occurrence leads to coordination between the neural and physiological systems underlying key-press and pupil responses, then we should observe coupling in their 1/f noise signals. However, reflexive pupil dilation is coordinated by the autonomic nervous system (ANS), whereas learned motor responses are coordinated by the central nervous system (CNS). These two physiological systems may not measurably be coupled when the body is at rest, as it is while sitting quietly during a tapping task.

For physiological fluctuations that were not responsive to the metronome, we measured heartbeat intervals. Resting heart rate should not be driven by the negligible effort required to execute each key-press. Yet healthy heartbeat intervals are known to exhibit 1/f noise (Peng et al., 1995), and both heart rate and pupil dilation are known to be coordinated by the ANS. Therefore, if the ANS is not driven by the tapping task, then we expect 1/f noise in pupil responses and heartbeat intervals to be coupled with each other, but independent of 1/f noise in key-press durations and timing deviations in tapping. The latter should be coupled with each other through the CNS and the tapping task.

### Experimental methods

#### Participants

Thirteen female and 13 male UC Merced students 18–30 years of age participated in the experiment for course credit or as volunteers. All reported having normal hearing and either normal or corrected vision. Four participants were left-handed. Data from two participants were removed due to equipment malfunction.

#### Apparatus

Pupil dilations were recorded using an Eye-Link II head mounted video-based eye-tracker (SR Research Ltd.) with a temporal resolution of 500 Hz and a spatial resolution of 0.025°. The eye-tracker uses two infrared LEDs mounted on the headband to illuminate each eye, and pupil dilations were recorded from whichever eye had the more accurate track. ECG samples were recorded at 250 Hz using a Zephyr™ Bioharness 3 (Zephyr Technology, Auckland, New Zealand) fastened around each participant as a chest belt. Taps were recorded using a keyboard and MAX 6 (Cycling 74) experiment software. The audiovisual metronome was presented using a 22-inch ThinkVision LCD monitor with 1280 × 1024 resolution, and Koss over-the-ear headphones. The metronome consisted of a 200 ms tone played at a loud but comfortable volume, synchronized with the display of a white circle for 200 ms with 25 cm diameter on a blank screen viewed from a 60 cm distance. A moderate level of light in the room was held constant across all participants.

#### Procedure

Participants were instructed to sit quietly for 10 min at the beginning of the experiment to allow the heart to settle to its resting rate. Participants were instructed how to fasten the Bioharness 3 to themselves, and the eye-tracker was calibrated using the standard nine-point calibration method. Participants were randomly assigned to either the synchronization condition (i.e., tapping in-phase with the metronome beats) or syncopation condition (i.e., tapping in between the metronome beats). Participants were instructed that they would see and hear a metronome beat presented at a constant, comfortable pace, and that they should tap the spacebar either in time with the beat or in between the beats. They were also instructed to keep their eyes fixated on the screen for the duration of the experiment. Each participant tapped to 1100 beats, which was set at a constant 800 ms inter-beat interval. This interval was set to be within the range of the healthy resting heart rate for young adults, and also to allow for 1100 beats to be administered in about 15 min.

#### Data pre-processing

The keyboard and heart rate apparatus directly produced time series of key-press durations and heartbeat intervals. Timing deviations were computed by subtracting each key-press time from its corresponding metronome beat. The interval timing of beats was known with high precision, but the phase of the metronome relative to key-press times was estimated for each participant. Any error in this estimate was constant across each time series of key-presses, and therefore not a factor.

The eye-tracker produced a sampled time series of pupil size that did not demarcate pupil responses to the flashes of light. However, pupil dilation responses could be seen as a clear waveform that rose and fell with roughly the same frequency as the metronome. We wrote a simple signal processing algorithm that found each peak value and trough value of the waveform. The algorithm iterated through the sampled time series from beginning to end, and determined “peak periods” and “trough periods” relative to prior minima and maxima. Each peak period started when the signal rose 100 units (approximately 5 μm per unit) above the previous minimum, and ended when the signal fell 100 units below its current peak value. Trough periods were defined conversely, and minima below half the previous maximum were discarded to remove eye blinks. The algorithm produced one time series of peak values and a corresponding time series of trough values for each participant. Analyses showed no qualitative difference in results between peak and trough time series, so here we report only analyses for peak dilation values.

The same trimming procedure was applied to all four time series for each participant: values above and below 2.5 standard deviations were removed. Then, if the remaining time series was shorter than 1024 measurements, it was padded with mean values to reach a length of 1024. If the remaining time series was longer than 1024, an even amount of beginning and ending values were trimmed to reach 1024 (with an extra value trimmed at the start for odd numbers).

## Results

Each individual time series was submitted to spectral analysis, and each resulting spectrum was logarithmically binned to create nine estimates of spectral power in nine evenly spaced frequency bins on a logarithmic scale (see also Thornton and Gilden, 2005). Logarithmic binning ensures that the same amount of data goes into each power estimate, and it also facilitates our spectral matching analyses reported below.

**Figure 1:**
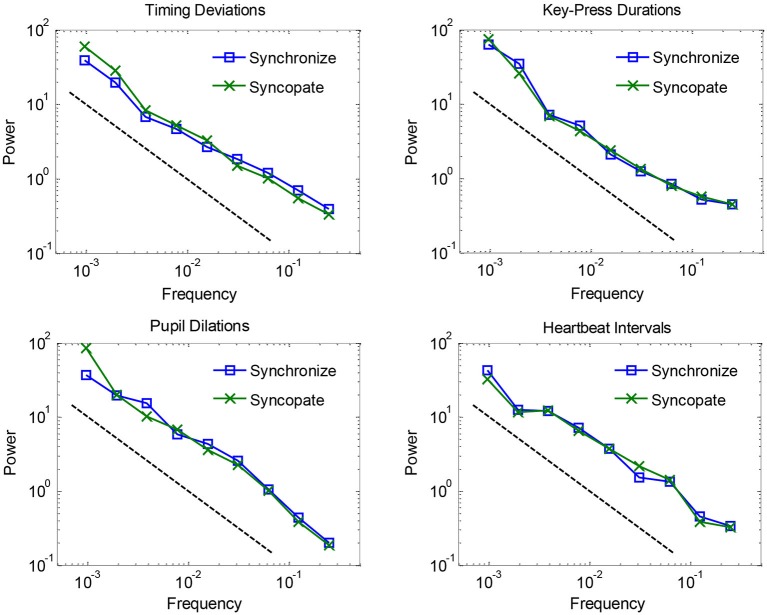
**Logarithmically binned spectra plotted for each of the four dependent measures, separated by synchronization vs. syncopation, and averaged across participants**.

Mean spectra are plotted in Figure 1 for each of the four dependent measures, separated by synchronization vs. syncopation. The graphs show that fluctuations for all measures in both conditions followed a clear 1/f scaling relation. 1/f exponents were estimated by fitting regression lines (and reversing their signs to account for the inverse relationship) to spectra for individual participants: mean values combining the two metronome conditions were 0.76 for timing deviations, 0.83 for key-press durations, 0.90 for pupil dilations, and 0.81 for heartbeat intervals, where 1.0 is ideal 1/f noise. Estimated exponents for the synchronization condition were not reliably different from the syncopation condition—all *t*-tests were within-subjects and had 12−1 = 11 degrees of freedom, and all yielded *t*-values less than 1, *t*_(11)_ < 1. Thus we did not replicate a previous study showing larger 1/f exponents for timing deviations when syncopating vs. synchronizing to a metronome (Chen et al., 2001). However, we found a trend in this direction (0.72 vs. 0.80, respectively), and we used an audiovisual metronome whereas the previous study used an audio-only metronome. Timing with pulsed visual signals is known to be less accurate than for audio signals (Chen et al., 2002), which might explain the small difference between our results and previous results (but see Hove et al., 2010). In any case, because there were no reliable effects of metronome condition, we combined them in subsequent analyses.

To test for coupling among 1/f noises, we used a measure of spectral convergence as an expression of complexity matching. In particular, log power estimates were subtracted per frequency bin for two given signals *a* and *b*, and the sum of their absolute values served as our measure of spectral convergence:

$$C_{a,b}=\sum_{f}\left|\log\left(S_{f,a}\right)-\log\left(S_{f,b}\right)\right|.$$

**Figure 2:**
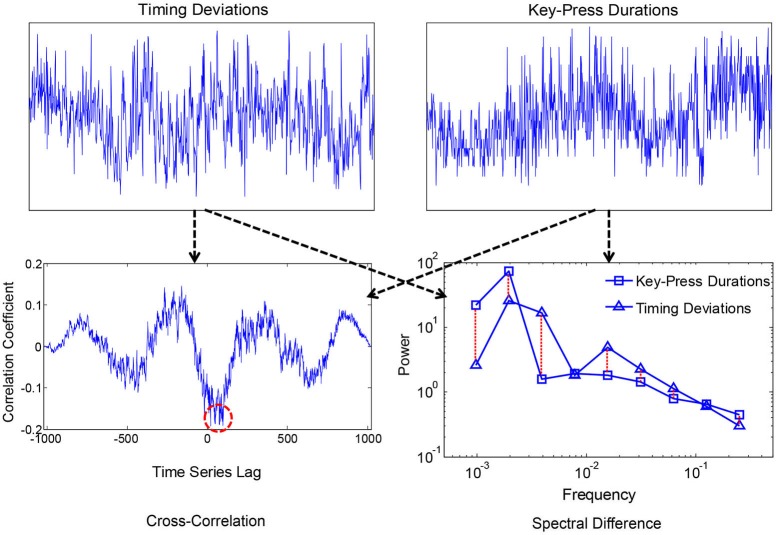
**Time series of peak key-press response times and durations for one participant (above), where the x-axis was the sequence of over 1000 key-presses, and the y-axis is normalized times and durations with 0 mean and showing +/−2.5 standard deviations**. Corresponding cross-correlation function and spectra are shown below. The red dashed circle shows the peak negative correlation, and the dashed lines between spectra show absolute log differences.

**Figure 3:**
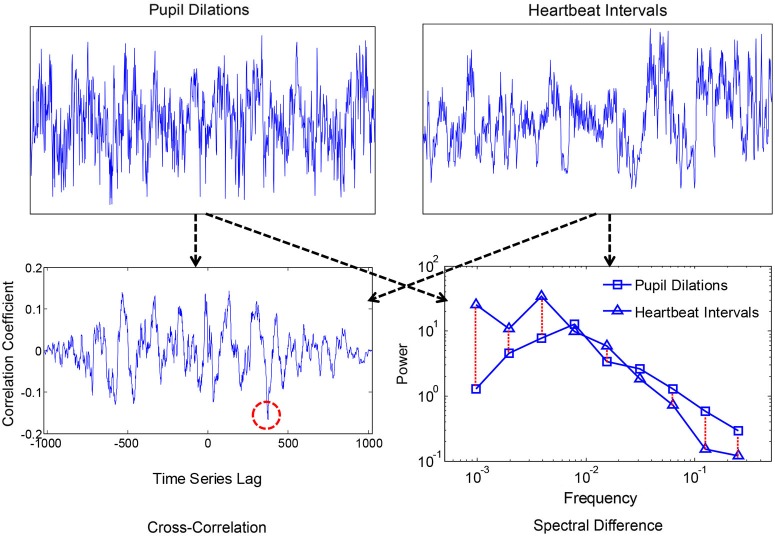
**Time series of pupil dilation responses and heartbeat intervals for one participant, along with the corresponding cross-correlation function and spectra**. The red dashed circle shows the peak negative correlation, and the red dashed lines between spectra show absolute log differences.

Smaller values corresponded with more similar i.e., convergent spectra. A measure of convergence was chosen over correlation of estimated 1/f exponents because the former is sensitive to idiosyncrasies in the individual 1/f-like spectra that converge towards 1/f in the average. Spectral Individual signals were compared because convergence is hypothesized to occur for the motor processes and ANS functions within individuals, as products of coordination, rather than across individuals. We also compared spectral convergence with cross-correlation to test whether coupling could be explained in terms of linear phase relations. Figures 2, 3 each show two example signals from one participant in the syncopation condition, along with two of the corresponding cross-correlation functions and two pairs of spectra to visualize their differences.

These measures of coupling cannot be interpreted without a baseline for comparison. With regards to spectral convergence, a spectral difference of zero is the absolute maximal similarity, but this measure does not have an inherent value or formula corresponding to chance similarity. We created baselines from surrogate pairings between signals from different participants. In particular, for each original comparison between time series *A* and *B*, a corresponding mean surrogate coupling was created by pairing each original time series with all *other* participants, i.e., 23 surrogate comparisons with *A* and 23 with *B*. Spectral convergence values were averaged for each set of 56 surrogate pairings to create a single baseline control for each pairing.

**Figure 4:**
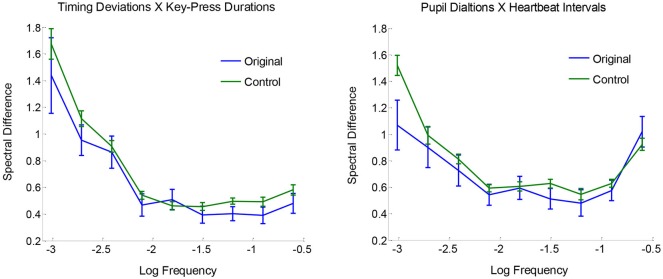
**Mean spectral differences |log(*S_*f,a*_*)− log(*S_*f,b*_*)| plotted as a function of frequency, for originals and surrogate controls, with standard error bars**.

Comparisons between original and surrogate coupling values showed a clear and consistent pattern of results: there was reliable spectral coupling between key-press timing deviations and key-press durations, and also between peak pupil dilations and heartbeat intervals. However, there were no reliable couplings across key-press and ANS measures. To examine the two observed effects of spectral convergence, Figure 4 plots the absolute log differences as a function of frequency, averaged for originals and baseline controls. Differences are generally greater in the lower frequencies, which appears to be attributable to more overall variability in spectral power relative to higher frequencies. Aside from this effect, original pairings are seen to be more similar to each other (i.e., smaller differences) compared with controls across the range of measured frequencies, indicative of coupling across timescales.

Statistical reliability of coupling was assessed using paired-samples *t*-tests with *C_a,b_* values for original pairings vs. their yoked controls. Spectra for timing deviations were reliably more similar to those for key-press durations compared with baseline controls, *t*_(23)_ = 2.52, *p* < 0.01, and the same was true for pupil dilations and heartbeat intervals, *t*_(23)_ = 2.18, *p* < 0.05. No other comparisons for spectral convergence approached significance, all *t*_(23)_ < 1. Mean *C_a,b_* values (with standard errors) for non-significant comparisons were the following for originals and controls, respectively: 0.83 (0.06) and 0.81 (0.02) for timing deviations X pupil dilations, 0.81 (0.05) and 0.81 (0.02) for timing deviations X heartbeat intervals, 0.88 (0.07) and 0.87 (0.03) for key-press durations X pupil dilations, and 0.87 (0.04) and 0.87 (0.02) for key-press durations X heartbeat intervals. Altogether, these tests show clear evidence of spectral convergence for key-press activity and for ANS activity, but not between the two.

Spectral convergence is not sensitive to the phase relation between the signals because phase information is discarded by spectral analysis. However, it is possible that phase relations played a role in the observed effects because two highly correlated signals (i.e., strong linear phase relation) will also have highly similar spectra. Here we show that signals may appear to be phase related, but that further analyses reveal these relations to be purely spectral in nature. Linear cross-correlation is perhaps the simplest and most common type of phase analysis, which tests for phase relations across the range of available lags. Given that we did not know a priori at what lag signals might be related, we simply took the peak *negative* correlation as a measure of maximal phase coupling. Preliminary analyses showed that peak negative correlations were slightly stronger than peak positive correlations, although both were weak: mean magnitudes varied within the small range of 0.15–0.21 across pairwise comparisons, at mean lags from about 140–340 beats apart. We report results with peak negative correlations, but results were not qualitatively different from peak positive correlations.

We conducted the same baseline control analysis as for spectral convergence, and we found the same pattern of effects as for spectral convergence: peaks were significantly more negative between timing deviations and key-press durations compared with baseline controls, *t*_(23)_ = 1.82, *p* < 0.05, and the same was true for pupil dilations and heartbeat intervals, *t*_(23)_ = 2.18, *p* < 0.05. And again, no other comparisons for spectral convergence approached significance, all *t*_(23)_ < 1.93, *p* < 0.05. A summary of the correlational and spectral coupling results is shown in Table 1, which contains mean differences between coupling measures for original pairings minus baseline controls, for all pairwise comparisons. The table shows that, for the two reliable comparisons, differences from baseline were proportionally greater for spectral coupling than for correlational coupling.

**Table 1:** **Mean correlational (top) and spectral (bottom) coupling effects for all pairwise comparisons between the four dependent measures (TD = Timing Deviations, KD = Key-Press Durations, PD = Pupil Dilations)**.

**Peak CC–Control**	TD	KD	PD
Timing Deviations			
Key-Press Durations	**0.036**		
Pupil Dilations	−0.001	0.017	
Heartbeat Intervals	0.001	−0.010	**0.032**
***C_a,b_*–Control**			
Timing Deviations			
Key-Press Durations	**0.829**		
Pupil Dilations	−0.126	−0.094	
Heartbeat Intervals	0.066	−0.025	**0.821**

*Statistically reliable effects are in bold, and the signs are reversed for correlational effects for consistent interpretation with spectral effects*.

These results suggest that simple linear phase relations may have contributed to the observed effects of spectral convergence, but it is curious that peak lags were so far apart. We do not know what type of phase coupling would explain phase relations offset by 2–4 min and well over 100 responses. An alternate possibility is that effects of spectral convergence can lead to spurious phase coupling when measured using our surrogate baseline analysis. We tested this alternative by using iterated amplitude adapted Fourier transform (IAAFT; Theiler et al., 1992; Schreiber and Schmitz, 1996), which scrambles phase relations in a given time series while preserving its spectral properties. If there is truly phase coupling, then cross-correlations for original comparisons should be stronger than those for the corresponding scrambled time series. Each surrogate pair had one original time series and one scrambled time series, and each original time series was paired with 100 scrambled series. We used paired-sampled *t*-tests to compare each original peak cross-correlation with the mean of its corresponding surrogate set.

Results from the IAAFT surrogate analysis revealed that there was no reliable linear phase coupling among any of the four dependent measures, as measured by peak cross-correlations. Surrogates were no different from originals for timing deviations and key-press durations, *t*_(23)_ = 1.5, *p* > 0.14, nor for pupil dilations and heartbeat intervals, *t*_(23)_ = 1.4, *p* > 0.17. The remaining comparisons were all near *t*_(23)_ ~ 1.4 or less. The IAAFT surrogate analysis provides additional evidence that the observed couplings in key-press responses and in measures of ANS functions (but not between the two) were expressed in terms of their power law spectral distributions, and not their phase relations (see also Kello et al., 2007).

## Discussion

The aim of the present experiment was to add to the body of evidence on the origins of 1/f noise in human timing, particularly with respect to two domain-general explanations. We employed a standard tapping task with synchronization and syncopation conditions, and we measured deviations in timing from a metronome. Our contribution was to record and analyze three additional repeated measures that varied in their relationship to timing deviations and the metronome. All four measures exhibited clear 1/f noise, consistent with previous studies suggesting that 1/f noise will manifest for any repeated measure of human behavior that is minimally perturbed and minimally constrained from one measurement to the next (Kello et al., 2010).

Our goal in eliciting these 1/f signals was to examine the relationships among them, as a way to test and elaborate upon the process summation vs. interdependent coordination accounts. The process summation account has served as a default explanation for many researchers over the years, in part because repeated measures of human timing and other behaviors might plausibly “pick up” on fluctuations in physiological and cognitive processes ranging across spatial and temporal scales of the brain and body. However, the idea of process summation has been called into question by a number of recent results. Our findings cast further doubt on this account because all four dependent measures exhibited distinct 1/f signals in terms of their phase relations—none were reliably cross-correlated relative to IAAFT controls. These findings are difficult to explain because at least some of these measures should pick up on the same summation of processes, which should result in reliable near-lag zero correlations. This is not what we found.

One could argue that each of our dependent measures tapped into a (mostly) distinct set of processes that each summed to produce distinct 1/f noises. However, while the 1/f signals had mostly unique phase profiles, their spectra were not fully distinct. Instead we found that spectra converged for timing deviations and key-press durations, and separately for pupil dilations and heartbeat intervals. These results indicate that 1/f fluctuations in different aspects of key-presses were coordinated across timescales, and likewise for ANS activity.

Interdependent coordination is in a better position to accommodate these results. We started with the basic premise that human timing is part and parcel with coordination, and that coordination requires a balanced, flexible coupling among whatever components are being coordinated. Flexible coupling is hypothesized to support the *soft-assembly* of sensorimotor function, and other types of biological and cognitive functions (Kello and Van Orden, 2009). A defining feature of highly adaptive systems is that their components can play multiple functional roles depending on context. In order to take on these different roles, components need to fall into different interdependent relationships under different conditions.

It is challenging to understand how biological and cognitive systems are so flexible. One valid and necessary approach is to study very particular examples and develop domain-specific theories to explain them. For instance, there are specific mechanisms of plasticity that re-organize sensorimotor maps in prism adaptation studies (Redding et al., 2005) or amputation cases (Sanes and Donoghue, 2000). However, it is equally valid and necessary to study basic principles from which many or even all mechanisms of sensorimotor function draw their flexibility. Metastability is one such principle that explicitly predicts 1/f noise to be a pervasive feature of systems of interdependent components poised near critical points. Theories of SOC have been formulated to explain why critical points appear to be so common to complex systems.

Metastability can explain 1/f noise in all four dependent measures, and it is consistent with the finding that two and only two pairs of these measures were coupled. However, the concept of metastability alone does not explain how spectral coupling can occur across timescales distinct from any phase coupling, nor does it explain the particular couplings of dependent measures that were observed. To explain the particular couplings observed, we will ultimately need domain-specific theories of manual sensorimotor control, and ANS function. For now, we can say that timing deviations and key-press durations measured two aspects of key-press dynamics that were coupled by the tapping task, and that coupling between pupil dilation and heartbeat intervals is “hard-wired” into the ANS. Moreover, tapping to a metronome while at rest did not enforce any physiological or informational demands on coupling between key-presses and the ANS. We conjecture that these systems would couple under more strenuous conditions, such as a sport with intense hand-eye coordination.

Finally, to explain spectral coupling across timescales, we refer to formal analyses of complexity matching that show maximal information exchange between complex systems with convergent power laws, yet distinct phase portraits. It is reasonable to assume that coordination is facilitated by maximal information exchange, and that key-press responses require information exchange among neural and motor processes involved in depressing and releasing the key on each response. It is also reasonable to assume that information must be exchanged among components of the ANS. As mentioned in the Introduction section, we do not mean information exchange in the sense of sending bits between components and subsystems. Instead we mean that components rely on each other to support sensorimotor and physiological functions (Kello and Van Orden, 2009). These functions are inherently multiscale, and hence the mutual interdependence that underlies them must span a range of spatial and temporal scales. Computational models based in metastability, such as critical branching networks (Kello, 2013), are needed to express formal theories of complexity matching in terms of neural, sensorimotor, and cognitive functions.

## Conflict of interest statement

The authors declare that the research was conducted in the absence of any commercial or financial relationships that could be construed as a potential conflict of interest.
